# BTG2 is a tumor suppressor gene upregulated by p53 and PTEN in human bladder carcinoma cells

**DOI:** 10.1002/cam4.1263

**Published:** 2017-12-13

**Authors:** Ke‐Hung Tsui, Kun‐Chun Chiang, Yu‐Hsiang Lin, Kang‐Shuo Chang, Tsui‐Hsia Feng, Horng‐Heng Juang

**Affiliations:** ^1^ Department of Urology Chang Gung Memorial Hospital‐Linkou Kwei‐Shan, Tao‐Yuan Taiwan; ^2^ Zebrafish center Department of General Surgery Chang Gung Memorial Hospital Keelung Taiwan; ^3^ Graduate Institute of Clinical Medical Science College of Medicine Chang Gung University Kwei‐Shan, Tao‐Yuan Taiwan; ^4^ Department of Anatomy College of Medicine Chang Gung University Kwei‐Shan, Tao‐Yuan Taiwan; ^5^ School of Nursing College of Medicine Chang Gung University Kwei‐Shan, Tao‐Yuan Taiwan

**Keywords:** Bladder cancer, BTG2, p53, PTEN, tumor suppressor gene

## Abstract

Although widely deemed as a tumor suppressor gene, the role of B‐cell translocation gene 2 (BTG2) in bladder cancer is still inconclusive. We investigated the role and regulatory mechanism of BTG2 in bladder cancer. BTG2 expression in human bladder tissues was determined by RT‐qPCR and immunoblotting assays. Expressions of BTG2 and PTEN in bladder carcinoma cells were determined by immunoblotting, RT‐qPCR, or reporter assays. The ^3^H‐thymidine incorporation assay, flow cytometry, and the xenograft animal model were used to determine the cell growth. BTG2 expression was lower in human bladder cancer tissues than normal bladder tissues. Highly differentiated bladder cancer cells, RT4, expressed higher BTG2 than the less‐differentiated bladder cancer cells, HT1376 and T24. Overexpression of BTG2 in T24 cells inhibited cell growth in vitro and in vivo. Camptothecin and doxorubicin treatments in RT‐4 cells or transient overexpression of p53 into p53‐mutant HT1376 cells induced p53 and BTG2 expression. Further reporter assays with site‐mutation of p53 response element from GGGAAAGTCC to GGAGTCC within BTG2 promoter area showed that p53‐induced BTG2 gene expression was dependent on the p53 response element. Ectopic PTEN overexpression in T24 cells blocked the Akt signal pathway which attenuated cell growth via upregualtion of BTG2 gene expression, while reverse effect was found in PTEN‐knockdown RT‐4 cells. PTEN activity inhibitor (VO‐OHpic) treatment decreased BTG2 expression in RT‐4 and PTEN‐overexpressed T24 cells. Our results suggested that BTG2 functioned as a bladder cancer tumor suppressor gene, and was induced by p53 and PTEN. Modulation of BTG2 expression seems a promising way to treat human bladder cancer.

## Introduction

Bladder cancer, the most commonly found urinary tract cancer, has around 350,000~380,000 new cases per year worldwide 1. There are two clinical phenotypes of bladder cancer: non‐muscle‐ and muscle‐invasive bladder cancers. Fifty to seventy percent of the non‐muscle‐invasive bladder cancers will recur and may turn into muscle‐invasive type with distant metastasis 2, which often lead the patient to a poor prognostic state 3. Thus, it is necessary to explore therapeutic targets for the treatment of bladder cancer.

B‐cell translocation gene 2 (BTG2) belongs to the anti‐proliferative (APR) gene family, which also include BTG1, BTG3, and Tob genes. TIS21, the homologous of BTG2, was first isolated from 3T3 fibroblasts 4. Then, the human BTG2 was cloned from chromosomal segment 1q32 5. The function of BTG2 in cancer growth inhibition has been previoulsy explored in several reports 6, 7, 8. Mao et al. further indicated that BTG2 caused G1 or G2/M cell cycle arrest dependent on the cell types 9. Our group has proved that BTG2 inhibited cell growth and induced either p53 dependently or independently in human prostate cancer cells 10. BTG2 has further identified as one of the prostate‐derived ets factor (PDEF) downstream genes in prostate cancer and bladder cancer cells 11, 12. However, the exact role of BTG2 in bladder cancer is still inconclusive. Hoffman et al. showed the raloxifene inhibitory effect on the RT4 cell growth via enhancement of BTG2 expression, suggesting BTG2 may play as a tumor suppressor gene in the bladder cancer 13. On the contrary, one report indicated that endogenous expression of BTG2 stimulated the migration of bladder cancer cell and higher BTG2 expression correlated with poor survival of patients with bladder cancer 14. Therefore, it is suggested further study to clarify the BTG2 role in bladder cancer.

Phosphatase and tensin homolog deleted on chromosome 10 (PTEN) has been widely known as a tumor suppressor gene and PTEN mutation or deletion is frequently noted in a lot of cancers 15. The most known function of PTEN is the negative regulator of PI3K/Akt/mTOR pathway, which is a crucial signal transduction pathway for cancer cell growth 16. For bladder cancer, loss of PTEN expression has been correlated with the disease invasiveness 17. However, the details regarding PTEN influences on the cell growth of bladder cancer and the PTEN downstream genes have not studied yet.

In this study, we investigated the roles of BTG2 and PTEN as well as the regulatory mechanisms of BTG2 in human bladder cancer. We aimed to provide new targets for bladder cancer therapy to improve the survival rate for the patients.

## Materials and Methods

### Cell cultures and chemicals

The bladder carcinoma cell lines RT4, HT1376, and T24 were purchased from the Bioresource Collection and Research Center (BCRC, Hsinchu, Taiwan, ROC) and maintained as described before 12. Fetal calf serum (FCS) was purchased from HyClone (Logan, UT), RPMI 1640 media was obtained from Invitrogen (Carlsbad, CA), Matrigel was purchased from BD Biosciences (Bedford, MA), and PTEN inhibitor, VO‐OHpic trihydrate, from Sigma (St. Louis, MO).

### Tissue collection and analysis

Tissues of human bladder comprised biopsy specimens obtained from patients admitted to the Department of Urology, Chang Gung University Hospital (Tao‐Yuan, Taiwan) and the protocol for tissue collection and analysis was approved by the Institutional Review Board of the Chang Gung Memorial Hospital (Approval: IRB 102‐3721B). Bladder tissues were classified based on the pathological examinations of the parallel preparations from respective samples by attending pathologists.

### Expression vector constructs and stable transfection

The amplified cDNA fragment containing the human BTG2 coding region was cloned into the eukaryotic expression vector pcDNA3 (Invitrogen) as described in detail previously 18. The human PTEN natural ORF mammalian expression plasmid (HG10421‐UT; pCMV3‐PTEN) was purchased from Sino Biological Inc. (Bejing, PR China). Electroporation was used to introduce expression vectors into the T24 cells, and the cells were selected with G418 (for T24‐BTG2) or hygomycin (for T24‐PTEN) as described in detail previously 12. The mock‐transfected T24 (T24‐DNA) cells were transfected with an empty expression vector (pcDNA3) and then clonally selected as the same as overexpressed cells.

### Knockdown p53 or PTEN

RT4 cells were transduced with lentiviral particles containing p53 shRNA (LVP343‐RB, GenTarget Inc., San Diego, CA) and PTEN shRNA (sc‐29459V, Santa Cruz Biotechnology, Santa Cruz, CA), respectively. Two days after transduction, RT4_shPTEN cells were incubated with 10 *μ*g/mL of puromycin dihydrochloride for at least three generations. Mock‐transfected cells (RT4_shCtrl) were transduced with control shRNA lentiviral particles (LVP‐Ctr‐RB, GenTarget Inc., and sc‐10808‐V, Santa Cruz Biotechnology, respectively) and clonally selected in the same manner as the knockdown cells.

### Immunoblotting assay

Equal quantities of cell extract were resolved in 10% SDS‐polyacrylamide gel and then transferred electrophoretically to a Hybond‐P PVDF membrane at 100 volts for 2 h. The membrane was first blocked with 10% skim milk (Sigma‐Aldrich) in TBS‐T, and then probed using antisera against BTG2 18, AKT (C67E7, Cell Signaling), pAKT (Ser473, Cell Signaling), GSK3*β* (12456; Cell signaling), Phospho‐GSK3*β* (5558; Cell signaling), mTOR (2983; Cell signaling), Phospho‐mTOR (2971; Cell signaling), p70S6K (9202; Cell signaling), Phospho‐p70S6K (9234; Cell signaling), or *β*‐actin antiserum (SC‐1616, Santa Cruz Biotechnology). Proteins were visualized using the Western Lightning Chemiluminescence Reagent Plus detection system (PerkinElmer, Inc., Waltham, MA). The ChemiGenius BioImaging System (Syngene, Cambridge, UK) was used to record band intensities, and the intensities were analyzed using the ChemiGenius GeneTool Program (Syngene).

### ^3^H‐thymidine incorporation assay

The ^3^H‐thymidine incorporation assay was used to measure cell proliferation as previously described 18.

### Flow cytometry

Cells were serum starved for 24 h and then cultured in RPMI 1640 medium with 10% FCS for another 24 h. Cell cycle analysis was performed using the FACS‐Calibur Cytometer and CellQuestPro Software (BD Biosciences); the data were analyzed using ModFit LT Mac 3.0 Software.

### Real‐time reverse transcription‐polymerase chain reaction (RT‐qPCR)

Total RNA was isolated using TRIzol^®^ reagent, and cDNA was synthesized using the Superscript III pre‐amplification system (Invitrogen). FAM^™^ dye‐labeled TaqMan^®^ MGB probes as well as PCR primers for human BTG2 (Hs00198887_m1), 18S (Hs03003631_g1), PTEN (Hs99999905_m1), and GAPDH (Hs99999905_m1) were purchased from Applied Biosystems (Foster City, CA). GAPDH (glyceraldehyde 3‐phosphate dehydrogenase; for the study of cells) and S18 (for the study of tissues) were used as internal positive probes. Real‐time reverse transcription‐polymerase chain reaction (RT‐qPCR) was performed and the mean cycle threshold (C_*t*_) values were calculated for internal control and target genes as described in detail previously 12.

### Reporter vector constructs and reporter assay

The human BTG2 (−297 to −1), reporter vectors were constructed as described in detail previously 11, 12. The mutant p53 response elements in BTG2 reporter vectors were constructed as described previously 18. Cells were seeded at a density of 10^4^ cells/well in a 24‐well plate and allowed to grow for 24 h. Cells were then transiently transfected with luciferase reporter vector for additional 48 h and relative luciferase activities were then measured and reported in relative light units (RLU) as previously described 19.

### Tumor xenograft study

The animal study has obtained approval from the Institutional Animal Care and Use Committee of the College of Medicine, Chang Gung University (IACUC Approval No.: CGU15092). Animal studies were performed in accordance with Laboratory Animal Facilities and Care guidelines (Council of Agriculture, Executive Yuan, Taiwan). Eighteen 4‐week‐old male BALB/cAnN‐Foxn1^NU^ mice were used in this study. Animals were purchased from the National Laboratory Animal Center, Taipei, Taiwan. Each mouse was anesthetized with a 100 *μ*L intraperitoneal injection of a mixture of 2.5% tribromoethanol and 2.5% tert‐amyl alcohol in Tris buffer solution. Prior prepared cancer cells (3 × 10^6^ cells/100 *μ*L) were mixed (1:1) with Matrigel and subcutaneously injected into one side of the back near the shoulder of each mouse. Mice were kept in a barrier facility under HEPA filtration and animal health was monitored twice per week during experiment. Xenograft growth was measured by Vernier calipers at intervals as indicated, and tumor volume was calculated using a previously described formula, namely Volume = [*π*/6 ×  largest diameter × (smallest diameter)^2^]^20^.

### Statistical analysis

Results are expressed as means ± SE of at least three independent experiments. Significant differences between groups were determined by one‐way ANOVA and the Student *t*‐test. All statistical analyses were carried out using the statistical package SigmaStat for Windows (Version 2.03, SPSS, Chicago, IL).

## Results

### Evaluation of BTG2 expression in human bladder cancer tissues and cell lines

**Figure 1:**
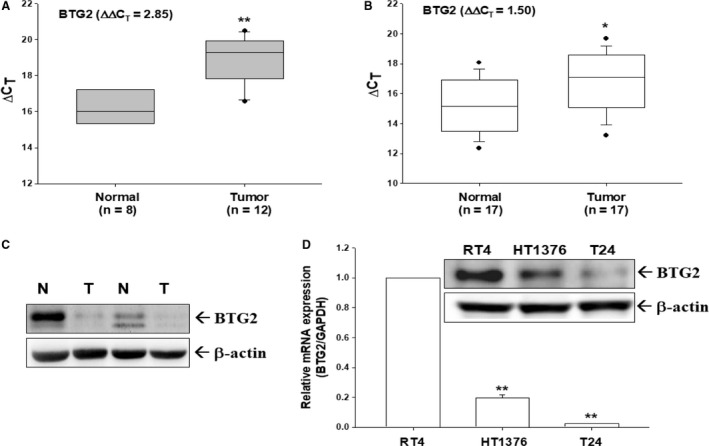
Evaluation of BTG2 expressions in human bladder tissues and carcinoma cells. (A) Quantitative analysis of BTG2 mRNA expression in unpaired bladder cancerous and normal tissues by RT‐qPCR. The result shown in a box plot. (B) Quantitative analysis of BTG2 mRNA expressions in paired bladder cancerous (white dot) and normal (black dot) tissues by RT‐qPCR. Solid and long dash lines represent the 95% confidence regression line of ΔCt of normal and cancerous tissues, respectively. (C) BTG2 protein expressions in two paired normal (N) and tumor (T) bladder tissues were determined by immunoblotting assays. Three kinds of bladder cells used in this study were serum starved for 24 h and subsequently incubated in RPMI media containing 10% FCS for another 24 h. (D) Cell proteins were then lysed for immunoblotting assay (top), and total RNA was extracted from cells for RT‐qPCR (bottom) assays. Data are presented as mean‐fold (± S.E.; *n* = 3) in relation to that of the RT4 cell group. (**P <* *0.05).

The mRNA expression of BTG2 was evaluated by RT‐qPCR. As shown in Figure 1A, BTG2 mRNA expression was higher in normal bladder tissues than cancerous tissues with the ∆∆CT of 2.85. Further measurement from paired normal and cancerous bladder tissues revealed that bladder cancer tissues presented with lower BTG2 mRNA expression (∆∆CT = 1.85, Fig. 1B) in comparison with bladder normal tissues. Results of immunoblotting assays also indicated that expression of BTG2 was lower in the cancer part than paired normal tissues (Fig. 1C). As compared to the highly differentiated bladder cancer cells, RT4, with other two less differentiated bladder cancer cells, HT1376 and T24, RT4 cells has higher BTG2 expression than HT1376 and T24 cells as determined by immunoblotting (Fig. 1D, top) and RT‐qPCR (Fig. 1D, bottom) assays.

### Evaluation of BTG2 role in human bladder cancer cell in vitro and in vivo

**Figure 2:**
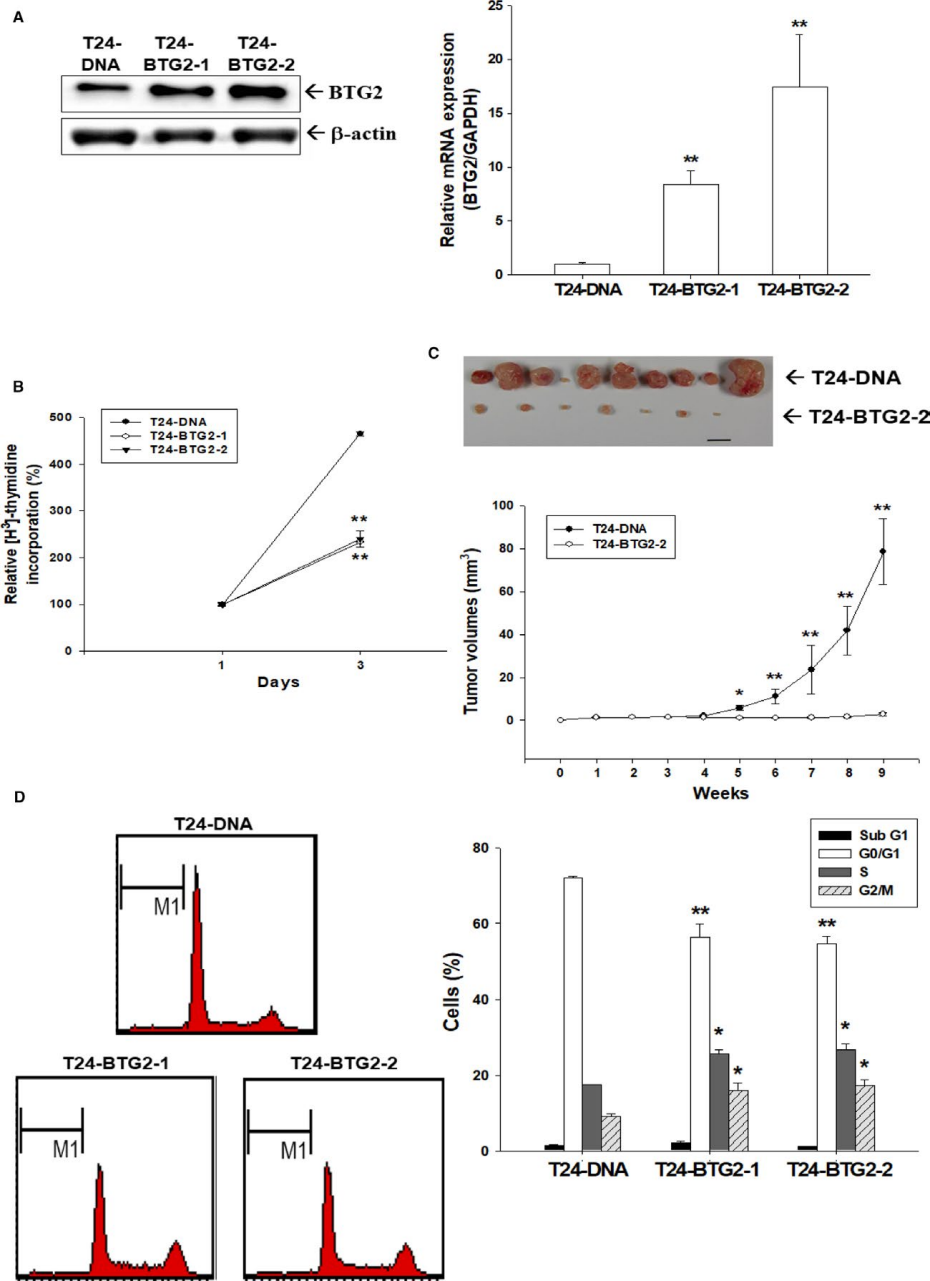
Effects of BTG2 on T24 cell proliferation in vitro and in vivo. Ectopic stably overexpression of BTG2 in T24 cells was confirmed by immunoblotting (A, left) and RT‐qPCR (A, right) assays. (B) Proliferative rates of T24‐DNA (black circle), T24‐BTG2‐1 (white circle), and T24‐BTG2‐2 (black triangle) cells were determined by ^3^H‐thymidine incorporation assays. Each point on the curve represents the mean percentage (± SE;* n* = 6) relative to that of day 1**.** (C) Nude mice were inoculated subcutaneously with T24‐DNA or T24‐BTG2‐2 cells. Tumor size was measured with vernier calipers and the data are presented as mean tumor size in mm^3^ (± SE;* n* = 9) at indicated time points. (D) The cell cycle distributions of T24‐DNA, T24‐BTG2‐1, and T24‐BTG2‐2 cells were analyzed after 24 h incubation by flow cytometry. The data shown in each bar chart represent the mean percentage ± SE (*n* = 3) of cells in each phase of cell cycle. (T24‐DNA: mock overexpression of BTG2 T24 cells; T24‐BTG2‐1 and T24‐BTG2‐2: BTG2 overexpression T24 cells) (**P <* *0.05).

To evaluate BTG2 role in human bladder cancer, we stably transfected BTG2 into T24 cells, and obtained T24‐BTG2‐1 and T24‐BTG2‐2 cells. As shown in Figure 2A, T24‐BTG2‐1 and T24‐BTG2‐2 cells presented higher BTG2 mRNA and protein expressions than T24‐DNA cells (T24 cells with mock BTG2 transfection). The cell proliferation of T24‐BTG2‐1 and T24‐BTG2‐2 cells were attenuated as compared with T24‐DNA cells as determined by ^3^H‐thymidie incorporation assay (Fig. 2B). In vivo animal study also revealed that xenografted T24‐BTG2‐2 cells grew much slowly than T24‐DNA cells (Fig. 2C). To investigate further the influences of BTG2 on T24 cell growth, the cell cycle distribution of T24‐DNA, T24‐BTG2‐1, and T24‐BTG2‐2 cells were evaluated by flow cytometry. Figure 2D demonstrated higher S and G2/M phase cells in both T24‐BTG2‐1 and T24‐BTG2‐2 cells. Collectively, our results suggested that BTG2 played as a tumor suppressor gene in human bladder cancer because BTG2 expressed lower in human bladder cancer tissues, and forced expression of BTG2 in human bladder cancer cells decreased cell growth in vitro and in vivo, which was partly attributed to cell cycle arrest induction at G2/M phase.

### p53 modulated B TG2 expression in human bladder cancer cells

**Figure 3:**
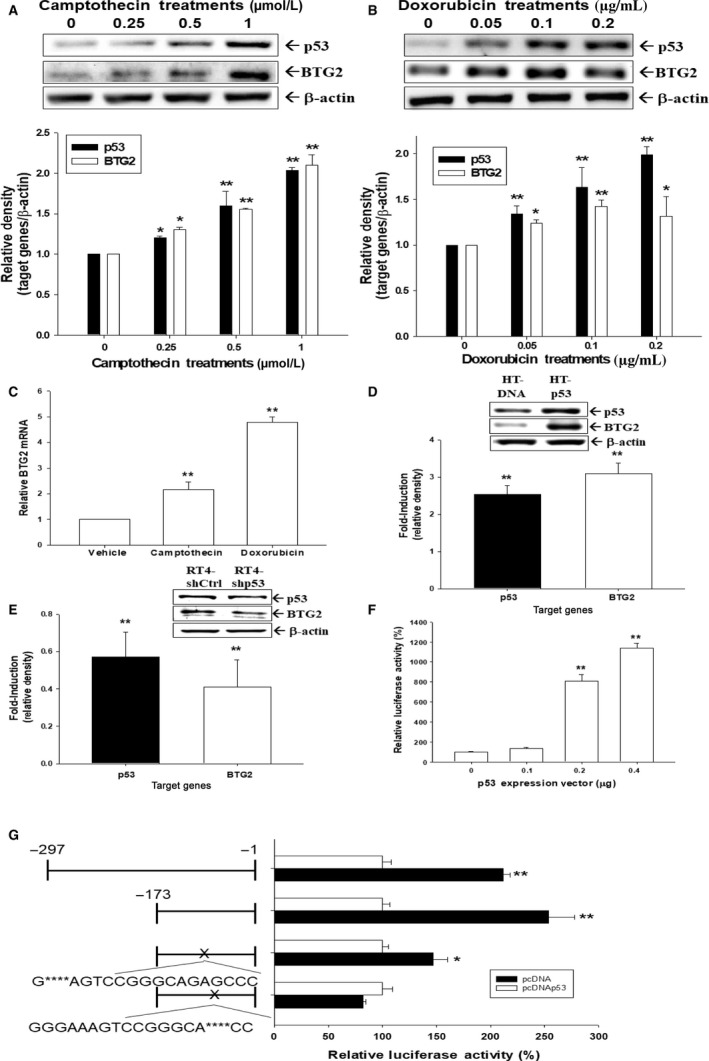
p53 upregulated BTG2 expressions in human bladder carcinoma cells. Expressions of BTG2 and p53 in RT4 cells following camptothecin (A) or doxorubicin (B) treatments were determined by immunoblotting assays. The quantitative analysis of immunoblotting assays was based on the intensity of the protein bands produced by the expressions of the target genes/*β*‐actin (± SE;* n* = 3) relative to the control solvent‐treated group. (C) Expressions of BTG2 in RT4 cells following 1 *μ*mol/L camptothecin or 0.2 *μ*g/mL doxorubicin treatment were determined by RT‐qPCR. The levels of BTG2 and p53 expressions of transiently ectopic p53‐expressed HT1376 cells (D) and p53‐knockdown RT‐4 cells (E) were determined by immunoblotting assays. (F) The activities of BTG2 report vectors, which were co‐transfected with various concentrations of p53 expression vectors into HT1376 cells for 72 h. (G) Luciferase activity of nested deletion or mutation constructs BTG2 reporter vectors‐transfected HT1376 cells after co‐transfected with pcDNA3 vector (white bars, mock transfection) or p53 expression vector (black bars). X represents the mutation of p53 response element. Data are expressed as the mean percentage ± S.E. (*n* = 6) of luciferase activity relative to mock‐transfected groups. (*P < 0.05, **P < 0.01). (HT‐DNA cells: HT1376 cells with mock overexpression of pcDNA3; HT‐p53 cells: HT1376 cells with p53 transient overexpression; RT4‐shp53: p53 knockdown RT4; RT4‐shCtrl: p53 mock knockdown RT4 cells).

Camptothecin (0.25–1 *μ*mol/L) and doxorubicin (0.05–0.2 *μ*g/mL) were applied to treat p53 wild‐type RT4 cells and both drugs induced p53 and BTG2 expression in RT4 cells dose‐dependently (Fig. 3A and B). Results of RT‐qPCR indicated that camptothecin (1 *μ*mol/L) and doxorubicin (0.2 *μ*g/mL) induced BTG2 mRNA expression in RT4 cells (Fig. 3C). HT‐p53 cells (HT1376 cells with p53 transient overexpression) has higher p53 and BTG2 expressions than HT‐DNA cells (HT1376 cells with mock overexpression of pcDNA3), while knockdown p53 in RT4 cells downregulated BTG2 expression (RT4‐shp53, p53 knockdown RT4; RT4‐shCtrl, p53 mock knockdown RT4 cells), which were determined by immunoblotting assays (Fig. 3D and E). The reporter assays revealed that BTG2 reporter activities were enhanced by treatments of p53 expression vectors in a dose‐dependent manner (Fig. 3F). Furthermore, reporter assays with 5′‐deletion and site‐mutation of p53 response elements from GGGAAAGTCC to GGAGTCC within BTG2 promoter area showed that the effect of p53 on BTG2 gene expression was dependent on the p53 response element (Fig. 3G).

### Evaluation of PTEN effect on cell growth and BTG2 mRNA expressions in human bladder cancer cells

**Figure 4:**
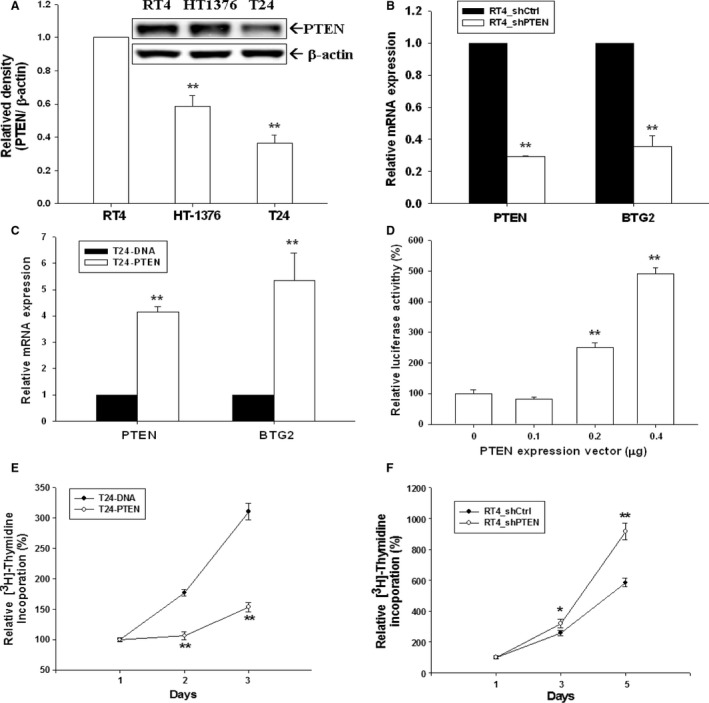
Evaluation of PTEN effects on BTG2 expression and on cell growth in vitro and in vivo in human bladder cancer cells. (A) The expressions of PTEN in three bladder cancer cell lines were determined by immunoblotting assays. The data are expressed as the intensity of the PTEN protein bands/*β*‐actin (± SE;* n* = 3) relative to the RT4 cells. The mRNA expressions of PTEN and BTG2 in PTEN‐knockdowned RT4 (RT4_shPTEN; white bars) cells, mock‐knockdowned RT4 (RT4_shCtrl; black bars) cells (B), PTEN‐overexpressed T24 (T24‐PTEN; white bars) and mock‐overexpressed T24 (T24‐DNA; black bars) cells (C) were determined by RT‐qPCR assays. Data are expressed as mean‐fold (± SE;* n* = 3) of the target genes relative to mock‐treated group. (D) BTG2 report vector was co‐transfected with various concentrations of PTEN expression vector into T24 cells for 72 h. Data are expressed as the mean percentage ± S.E. (*n* = 6) of luciferase activity relative to mock‐transfected groups. (**E**) The rates of cellular proliferation in T24‐DNA cells and T24‐PTEN cells were analyzed by ^3^H‐thymidine incorporation assays. (**F**) The rates of cellular proliferation in RT_shCtrl cells and RT4_shPTEN cells were analyzed by ^3^H‐thymidine incorporation assays. (*P < 0.05, **P < 0.01).

PTEN expression was evaluated in RT4, HT1376, and T24 cells with highest and lowest PTEN expressions in RT4 and T24 cells, respectively (Fig. 4A). To investigate PTEN effect on human bladder cancer, PTEN was knocked down or overexpressed in RT4 (Fig. 4B) or T24 (Fig. 4C) cells, respectively. The BTG2 expressions were decreased by PTEN knockdown in bladder cancer cells as RT4_shPTEN cells (RT4 cells with PTEN knockdown) exhibited lower BTG2 mRNA expression than RT4_shCtrl cells (RT4 cells with mock knockdown) (Fig. 4B); while T24‐PTEN cells (T24 cells with PTEN overexpression) presented higher BTG2 mRNA expression than T24‐DNA cells (T24 cells with mock overexpression) (Fig. 4C). The result was further supported by the reporter assays, which showed that BTG2 reporter activities were increased in a dose‐dependent manner as treated by PTEN expression vectors (Fig. 4D). Figure 4E shows that T24‐PTEN cells had lower cellular proliferation rate than T24‐DNA cells; while RT4_shPTEN cells exhibited higher cell proliferation rate than RT4_shCtrl cells (Fig. 4F). Collectively, our results indicated that PTEN repressed cell growth of the bladder cancer in vitro, and negatively modulated BTG2 mRNA expression in bladder cancer cell.

### Evaluation of PTEN downstream signals and genes in human bladder cancer cells

**Figure 5:**
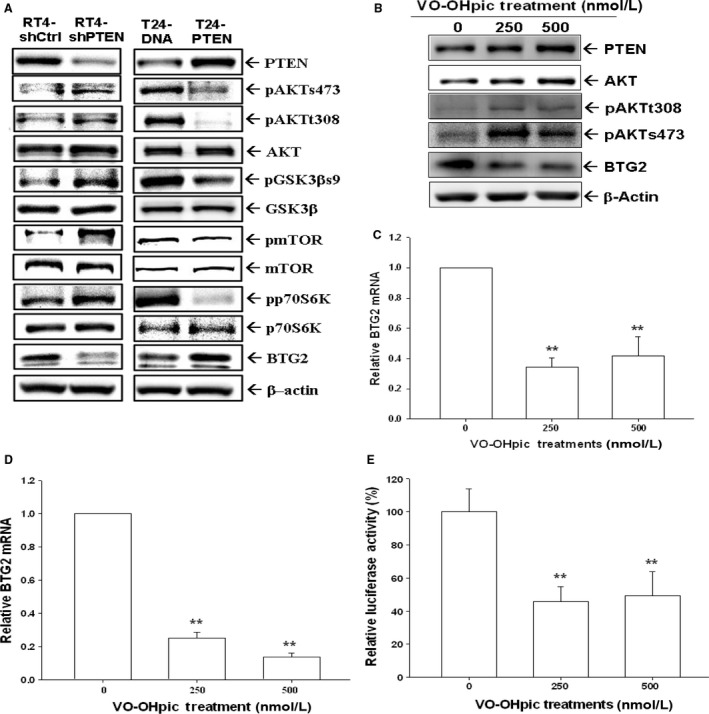
Effects of PTEN modulation on downstream signal transductions and BTG2 in human bladder cancer cells. (A) The expressions of PTEN, pAKTs473, pAKTt308, AKT, pGSK3b, GSK3b, pmTOR, mTOR, P70S6K, pP70S6K, and BTG2 in T24‐DNA and T24‐PTEN cells (left), and in RT4_shCtrl and RT4_shPTEN (right) were determined by immunoblotting assays. (B) RT4 cells were treated with various dosages of VO‐OHpic trihydrate. Expressions of PTEN, Akt, p‐Akt (t308 and s473), BTG2, and *β*‐actin were determined by immunoblotting assays. Expressions of BTG2 mRNA in RT4 (C) and PTEN‐overexpressed T24 (D) cells following various concentrations of VO‐OHpic trihydrate treatments were determined by RT‐qPCR assays. (E) The BTG2 reporter vector‐transfected T24‐PTEN cells were treated with various concentrations of VO‐OHpic trihydrate for 24 h. Data are expressed as the mean percentage ± S.E. (*n* = 6) of luciferase activity relative to solvent‐control groups. (**P < 0.01).

We further evaluated PTEN downstream signals expressions in bladder cancer cells. T24‐PTEN cells showed lower pAKTs473, pAKTt308, pGSK3b, pmTOR, and pP70S6K expressions than T24‐DNA cells; while RT4_shPTEN cells presented higher pAKTs473, pAKTt308, pGSK3b, pmTOR, and pP70S6K expressions than RT4_shCtrl cells (Fig. 5A). Figure 5A demonstrated that PTEN increased BTG2 protein expression in human bladder cancer cells as T24‐PTEN cells exhibited higher BTG2 expression than T24‐DNA cells; while RT4_shPTEN cells revealed lower BTG2 expression than RT4_shCtrl cells. Then, we treated RT4 cells with VO‐OHpic trihydrate, one kind of PTEN activitiy inhibitor, and the expression of p‐Akt (t308 and s473) was increased, but BTG2 was decreased while PTEN and Akt expressions remained the same (Fig. 5B). The BTG2 mRNA expression was inhibited by VO‐OHpic trihydrate in RT4 cells (Fig. 5C) and T24‐PTEN (Fig. 5D) cells. The reporter assay for BTG2 reporter vector‐transfected T24‐PTEN cells treated by varied concentrations of VO‐OHpic trihydrate revealed that the BTG2 reporter activity was decreased by VO‐OHpic trihydrate (Fig. 5E). Collectivley, our results indicated that BTG2 expression in human bladder cancer cells was stimulated by PTEN.

## Discussion

In this study, we demonstrated that BTG2 served as a tumor suppressor gene in human bladder cancer in vitro and in vivo and lower BTG2 expression was found in human bladder cancer tissues as compared to normal bladder tissues. The expressions of BTG2 were stimulated by p53 and PTEN in human bladder cancer cells. PTEN deficiency also enhanced cell growth of the human bladder cancer. Our results suggested that modulation of BTG2 expression is a new therapeutic direction for human bladder cancer.

BTG2 belongs to the BTG/TOB anti‐proliferative proteins family, besides BTG2, which also comprises BTG1, BTG3, BTG4, TOB1, and TOB2 featuring the conserved N‐terminal BTG domain 21, 22. Although widely deemed as a tumor suppressor gene, the role of BTG2 in human bladder cancer has not disclosed fully with higher BTG2 expression associated with reported poor prognosis of human bladder cancer patients 14. Our results indicate that human bladder cancer exhibited lower BTG2 mRNA and protein expression as compared to normal bladder tissues (Fig. 1A, B, and C). The higher differentiated human bladder cancer cells, RT4, possessed higher BTG2 protein expression than other two less differentiated human bladder cancer cells, HT1376 and T24 (Fig. 1D). To understand BTG2 role in bladder cancer, BTG2 was then transfected into T24 cells. Figure 2B demonstrated that both T24‐BTG2‐1 and T24‐BTG2‐2 cells presented with slower proliferative rate as compared with T24‐DNA cells. The xenografted T24‐BTG2‐2 tumor exhibited smaller tumor volume than T24‐DNA cell group (Fig. 2C). Collectively, since BTG2 presented with higher expression in normal bladder tissues than bladder cancer tissues and forced expression of BTG2 in human bladder cancer cells inhibited cancer cell growth in vitro and in vivo, we thus concluded that BTG2 played as a tumor suppressor gene in human bladder cancer.

Cell cycle progression is the necessity for cell to proliferate and is under well orchestration and strict control to maintain human tissue homeostasis. The uncontrolled cell proliferation of cancer mainly can be attributed to the cell cycle deregulation 23. Thus, cell cycle progression emerges as a good target for cancer treatment. BTG2 was proposed as a pan‐cell cycle regulator before 24, which could induce G1/S or G2/M arrest in a tissue‐ or cell‐specific manner. Previous studies indicated that overexpression of BTG2 induces growth inhibition of 293 and OSCC cells by modulation of cyclin A, cyclin B, cyclin D1, or cyclin E 25. As we analyzed cell cycle distribution of T24‐DNA, T24‐BTG2‐1, and T24‐BTG2‐2 cells by flow cytometry, higher G2/M and S phase cell percentages were found in T24‐BTG2‐1 and T24‐BTG2‐2 cells (Fig. 2D), indicating BTG2 transfection could induce G2/M arrest in human bladder cancer cells, leading to the growth inhibition found in Figure 2B and C.

p53 is a well‐known tumor suppressor gene and p53 mutations have been identified in a variety of human cancers 26. Previously, p53 response element has found to exist within BTG2 promoter area, indicating BTG2 expression is modulated by p53 5, 10, 27. To investigate whether BTG2 expression is modulated by p53 in human bladder cancer cells, camptothecin (0.25–1 *μ*mol/L) and doxorubicin (0.05–0.2 *μ*g/mL) were applied to treat p53 wild‐type RT4 cells. Figure 3A, B and C demonstrated that both drugs could induce p53 and BTG2 expressions in RT4 cells dose‐dependently. Transient overexpression of p53 in p53‐null HT1376 cells increased both p53 and BTG2 expressions (Fig. 3D), while knockdown p53 in p53‐wild‐type RT4 cells decreased both p53 and BTG2 expressions (Fig. 3E), which was also supported by the increased BTG2 reporter activity as treated by varied doses of p53 expression vectors in HT1376 cells (Fig. 3F). The reporter assays with 5′‐deletion and site‐mutation of p53 response elements within BTG2 promoter area further confirmed that p53 induced BTG2 gene expression through interacting with the p53 response element located at BTG2 promoter area (Fig. 3G). Taken together, our results indicated that BTG2 was stimulated by p53 in human bladder cancer cells. These results are consistent with our previous studies in the prostate carcinoma cells 18, 27. Besides p53, several reports has indicated that JNK, ERK, p38, NF*κ*B, WNT/*β*‐catenin, AKt/sp1/NOx4, and Src/FAK pathways also modulate BTG2 expressions in different cancer cells 28, 29, 30, 31, 32. PTEN, identified in 1997 in chromosome 10q23, is a well‐known tumor suppressor gene. The finding of the late stage cancer usually has inactivated PTEN renders PTEN a hot issue for cancer treatment research in the past decades 33, 34. The main function of PTEN lies in the negative regulation of PI3K/Akt/mTOR pathway, which plays a vital role in regulating many important signaling pathways, which mainly induce cell growth and metastasis 15. As we evaluated PTEN expressions in three kinds of human bladder cancer cells, RT4, the mostly differentiated cells among these three kinds of cancer cells, presented with the highest PTEN expression (Fig. 4A). As we knocked down or overexpressed PTEN, the phosphorylation of downstream signal proteins were changed, including pAKTs473, pAKTt308, pGSK3b, pmTOR, and pP70S6K (Fig. 5A). The findings of that T24‐PTEN cells had lower cell proliferative rate than T24‐DNA cells and RT4_shCtrl had lower cell proliferative rate as compared to RT4_shPTEN cells demonstrated the tumor suppressor gene role of PTEN in human bladder cancer cells (Fig. 4E and 4F). Since early study indicated that PTEN and BTG2 were canonical downregulated by miR‐21 overexpression in myeloma cells (Leone et al., 2013), we further demonstrated that PTEN also stimulated BTG2 in human bladder cancer. Ectopic overexpression PTEN could increase BTG2 expression while knockdown PTEN or PTEN activity inhibitor treatment could decrease BTG2 expression (Fig. 4 and 5). Thus, we concluded that PTEN insufficiency would increase cell growth of the human bladder cancer with BTG2 positively regulated by PTEN. Previous study has indicated that PTEN induced p53 acetylation which regulated p53 protein stability in osteosarcoma U2OS cells 35. Whether PTEN induced p53 protein stability to upregulate BTG2 gene expression in bladder carcinoma cells needs further investigation. However, based on our study showing ectopic overexpression of PTEN in the p53‐null T24 cells induced BTG2 expression, PTEN upregulated BTG2 expression is, in part, not via the p53 signal pathway in bladder cancer cells (Fig. 4C).

## Conclusion

Although most bladder cancer patients diagnosed in the early stage, still 70% of patients have cancer recurrence and 10% of the recurrent patients have bladder cancer with muscle involvement, which has the high possibility of concurrent distant metastasis, leading to the poor prognosis 36. Thus, to find out more targets for bladder cancer treatment is warranted. Our current work demonstrated that BTG2 functioned as a tumor suppressor gene in human bladder cancer and induced by p53 and PTEN. PTEN served as a tumor suppressor gene as well in human bladder cancer. Our results suggest that modulation of BTG2 expression is a promising direction for bladder cancer treatment.

## Conflict of Interest

All authors declare that there are not completing interest.
